# The Mirror Neurons Network in Aging, Mild Cognitive Impairment, and Alzheimer Disease: A functional MRI Study

**DOI:** 10.3389/fnagi.2017.00371

**Published:** 2017-11-15

**Authors:** Elisabetta Farina, Francesca Baglio, Simone Pomati, Alessandra D'Amico, Isabella C. Campini, Sonia Di Tella, Giulia Belloni, Thierry Pozzo

**Affiliations:** ^1^Neurorehabilitation Unit, IRCCS S. Maria Nascente, Don Gnocchi Foundation, Milan, Italy; ^2^INSERM-U1093, Cognition-Action-Plasticité sensorimotrice, Campus Universitaire, Dijon, France; ^3^Neuroimaging Unit, IRCCS S. Maria Nascente, Don Gnocchi Foundation, Milan, Italy; ^4^Neurology Unit, Luigi Sacco Hospital, Università degli Studi di Milano, Milan, Italy; ^5^Centro di Neurofisiologia traslazionale, Istituto Italiano di Tecnologia, Ferrara, Italy

**Keywords:** mirror neurons, Alzheimer disease, Mild Cognitive Impairment, aging, fMRI, neuropsychological tests

## Abstract

The aim of the current study is to investigate the integrity of the Mirror Neurons (MN) network in normal aging, Mild Cognitive Impairment (MCI), and Alzheimer disease (AD). Although AD and MCI are considered “cognitive” diseases, there has been increasing recognition of a link between motor function and AD. More recently the embodied cognition hypothesis has also been developed: it postulates that a part of cognition results from the coupling between action and perception representations. MN represent a neuronal population which links perception, action, and cognition, therefore we decided to characterize MN functioning in neurodegenerative cognitive decline. Three matched groups of 16 subjects (normal elderly-NE, amnesic MCI with hippocampal atrophy and AD) were evaluated with a focused neuropsychological battery and an fMRI task specifically created to test MN: that comprised of an observation run, where subjects were shown movies of a right hand grasping different objects, and of a motor run, where subjects observed visual pictures of objects oriented to be grasped with the right hand. In NE subjects, the conjunction analysis (comparing fMRI activation during observation and execution), showed the activation of a bilateral fronto-parietal network in “classical” MN areas, and of the superior temporal gyrus (STG). The MCI group showed the activation of areas belonging to the same network, however, parietal areas were activated to a lesser extent and the STG was not activated, while the opposite was true for the right Broca's area. We did not observe any activation of the fronto-parietal network in AD participants. They did not perform as well as the NE subjects in all the neuropsychological tests (including tests of functions attributed to MN) whereas the MCI subjects were significantly different from the NE subjects only in episodic memory and semantic fluency. Here we show that the MN network is largely preserved in aging, while it appears involved following an anterior-posterior gradient in neurodegenerative decline. In AD, task performance decays and the MN network appears clearly deficient. The preservation of the anterior part of the MN network in MCI could possibly supplement the initial decay of the posterior part, preserving cognitive performance.

## Introduction

In the current demographic context, aging, and neurodegenerative diseases linked to aging, have become a very important social issue. Alzheimer Disease (AD), the most common form of dementia, is a neurodegenerative disease. The incidence of AD grows exponentially in aging. As actually there is no cure, several studies are focusing on prevention. A category which now represent a preferential target of intervention is Mild Cognitive Impairment (MCI). As originally defined, MCI was characterized primarily as an amnestic disorder that represented an intermediate stage between normal aging and AD (Petersen et al., 1999). More recently, broader conceptualizations of MCI have emerged that also encompass cognitive domains other than memory. The term amnestic MCI (aMCI) was recommended for individuals in the predementia phase prior to AD as a separate group from those with MCI preceding other forms of dementia (Petersen et al., 2001; Petersen and Morris, 2005). Even if MCI definition has been criticized (the degree of functional preservation is sometimes difficult to define), MCI is a useful clinical concept because it is a well-recognized risk factor for dementia (Busse et al., 2003; Ward et al., 2013) and therefore, represents a critical window of opportunity to intervene and alter the trajectory of both cognitive and functional decline in seniors.

Even if AD and MCI have been characterized as “cognitive” diseases until now, over the last decade there has been increasing recognition of a link between motor function and the risk of developing AD. Both a lower level and more rapid rate of motor decline in cognitively intact individuals predicts the subsequent development of MCI and AD, and loss of motor function can precede cognitive impairment by several years (Verghese et al., 2008; Buchman and Bennett, 2011). Moreover, physical activity is recurrently associated to lower incidence of dementia and to better cognition in affected patients (Groot et al., 2016; Ginis et al., 2017). Hand and facial movement training has been used in people with MCI, leading to improvement of executive functions (Scherder et al., 2005). Hand movements are known to stimulate areas in the frontal lobe engaged in sensorimotor and cognitive processes (Binkofski and Buccino, 2006; King et al., 2014). In this context, the study of the Mirror Neurons (MN) could be of particular interest, as a neuronal population that links perception and action and also cognition and motility. The MN represent a distinctive class of neurons that discharge both when an individual executes a motor act and when he observes another individual performing the same or a similar motor act. They were first discovered in a monkey's brain, in particular in the ventral premotor area F5 and in the inferior parietal lobule (Di Pellegrino et al., 1992; Rizzolatti et al., 2014). In humans, brain activity consistent with that of MN has been found in the primary motor cortex (M1; Fadiga et al., 2005), the premotor cortex (posterior regions of the inferior frontal gyrus—IFG—, considered the human homolog of the monkey F5) (Kilner et al., 2009; Ferri et al., 2015), and in the inferior parietal lobule (IPL) (Rizzolatti et al., 2001; Rizzolatti and Craighero, 2004; Chong et al., 2008; Arnstein et al., 2011). The MN presence in humans has been studied through neuroimaging (fMRI) and neurophysiological techniques. fMRI experiments have shown that a fronto-parietal network, remarkably similar in monkeys and humans, is activated during observation and execution of hand grasping acts, as well as during observation of the act of grasping tools (see Molenberghs et al., 2012 for a review). However, many neuroimaging studies are based on action observation alone, rather than on a comparison between action observation and execution (Turella et al., 2009b). Among the fMRI studies looking at the true action observation execution mechanism in humans, Gazzola et al. (2007) found that the MN system was activated by observing both human and robotic actions, only if the robotic action was not repetitive. According to their findings, the goal of an action might be more important for mirror activation, than the way in which the action is performed. Turella et al. (2009a), came to similar conclusions; they investigated whether the “mirror” regions activity was modulated by the type of view and thus responded in a similar fashion to the observation of an isolated hand or multiple actions of a person. The MN system responded in the same way in all the observed conditions, thus supporting the hypothesis that the action goal is one of the key areas for the observation of MN activity.

The discovery of MN has given a biological substrate to the simulation theory. This is the relatively recent idea, that actions involve both an overt and a covert stage, the latter being a sort of dynamic cognitive representation of the future. This includes the goal of the action, the means to reach it, and to predict its consequences (Jeannerod and Frak, 1999; Jeannerod, 2001). A development of this theory is the model of the “Embodied Cognition” that combines thinking and acting. This is an antidote to the traditional division between cognition, perception and action (Barsalou, 1999; Wilson, 2002; Glenberg, 2010; Willems and Francken, 2012). MN are now thought responsible for sophisticated human behavior and thought processes such as language (Rizzolatti and Arbib, 1998; Tettamanti et al., 2005; Fischer and Zwaan, 2008; Chwilla et al., 2011; Pulvermüller et al., 2014), empathy (Decety and Jackson, 2004; Iacoboni, 2009; Corradini and Antonietti, 2013), motor memory (Stefan et al., 2005), and imitation (Bonini and Ferrari, 2011; Oh et al., 2012).

Characterizing the functioning of the MN network in neurodegenerative diseases would thus be useful to the better understanding of functional mechanisms underlining clinical manifestations. It would also allow to capitalize on these kinds of neurons in the rehabilitation of motor and cognitive symptoms. There have been some theoretical proposals about interpreting neurodegenerative diseases, in particular Amyotrophic Lateral Sclerosis and Frontotemporal Dementia, as linked to dysfunction of perception-action circuits including the MN network (Bak, 2013; Taylor et al., 2013; Eisen et al., 2014). However, direct studies on MN functioning through neuroimaging or neurophysiological techniques are scarce. Some results on the MN network, even if still controversial, are available for Parkinson disease (Tremblay et al., 2008; Albert et al., 2010; Alegre et al., 2010, 2011; Pelosin et al., 2013; Heida et al., 2014). Only recently, a seminal paper (Moretti, 2016) was published, where 74 adult subjects with MCI underwent EEG recording and a high resolution morphological MRI. This paper gives support to the hypothesis that a possible pathological uncoupling of the MN system with malfunctioning of the IPL areas could explain the cognitive deficits in prodromal AD. As far as a possible rehabilitation use is concerned, Eggermont et al. (2009) is the only published study that focuses on utilizing the MN system to improve cognitive performance in people with AD. However, prior to the consideration of the use of this neuronal system in rehabilitation, it would be necessary to evaluate the status of conservation, i.e., if it is decreased or increased in neurodegenerative cognitive impairment.

The aim of the current study is to investigate the integrity of the MN network in normal aging, MCI and AD. For this purpose, three matched groups of subjects (normal elderly people, people affected by aMCI and AD patients) were evaluated with an fMRI task specifically constructed to test the MN circuitry (Cabinio et al., 2010).

On the basis of previous research, we anticipated the activation of the frontal cortex (Brodmann area BA6 and precentral gyrus,) the parietal cortex (rostral part of the inferior parietal lobule, BA40) and possibly the temporal cortex (superior temporal gyrus BA22 and BA42) if the MN network, and the associated motor resonance mechanism, in the groups tested, were preserved. However, it would also be possible to observe an increased activation of areas belonging to the MN system or linked to it in the MCI group, as a functional adaptation process, to counterbalance the neural damage, in the same way that it has been described in mild Multiple Sclerosis patients (Filippi et al., 2004; Pierno et al., 2009).

## Materials and methods

### Subjects

Sixteen AD patients and an equal number of aMCI subjects and aged healthy subjects (NE) were enrolled and performed an fMRI study and a neuropsychological assessment.

Table 1 summarizes the demographic data of the three groups. Age, education level, and gender was not significantly different (ANOVA). Probable AD was diagnosed according to NIA-AA criteria (McKhann et al., 2011). aMCI subjects were diagnosed according to Petersen's criteria (Petersen et al., 1999, 2001). Only MCI subjects with significant atrophy of at least one hippocampus were included in the study (see later). The following is the exclusion criteria: patients unable to understand and/or follow instructions; severe attentional deficits, untreated psychiatric disorders, joint deformity of arthritic origin, visual or hearing deficits, previous stroke or other neurological disorders, contraindications to MRI. Persons with MCI or dementia were consecutively recruited from the Don Carlo Gnocchi Foundation Service for Cognitive Disorders and Dementia. Patients were diagnosed after taking their clinical history and carrying out medical and neurological examinations, routine blood tests and neuropsychological assessment. All patients also underwent either brain CTs or MRIs to evaluate the vascular lesion load and to exclude rarer causes of dementia (e.g., tumors, hematomas, etc.). The control group consisted of spouses or hospital volunteers.

**Table 1:** Demographic data of the three groups (ANOVA)^*^.

	**NE**	**MCI**	**AD**	***p***
#	16	16	16	
Age	73.8 ± 6.8	77.0 ± 4.8	77.8 ± 5.4	NS
Level of education (Primary, Secondary, High Scool, University)	0\3\8\5	2\3\9\2	5\5\4\1	NS
Gender: F\M (F%)	9\7 (56.3)	6\10 (37.5)	9\6 (60)	NS

* *Non-parametric tests (Kruskal—Wallis) give the same results*.

*NE, Normal Elderly people; MCI, Mild Cognitive Impairment; AD, Alzheimer Disease*.

This study was carried out in accordance with the Declaration of Helsinki. All subjects gave written informed consent. The protocol was approved by the Don Gnocchi Foundation ethical hospital committee.

### fMRI experimental paradigm

**Figure 1:**
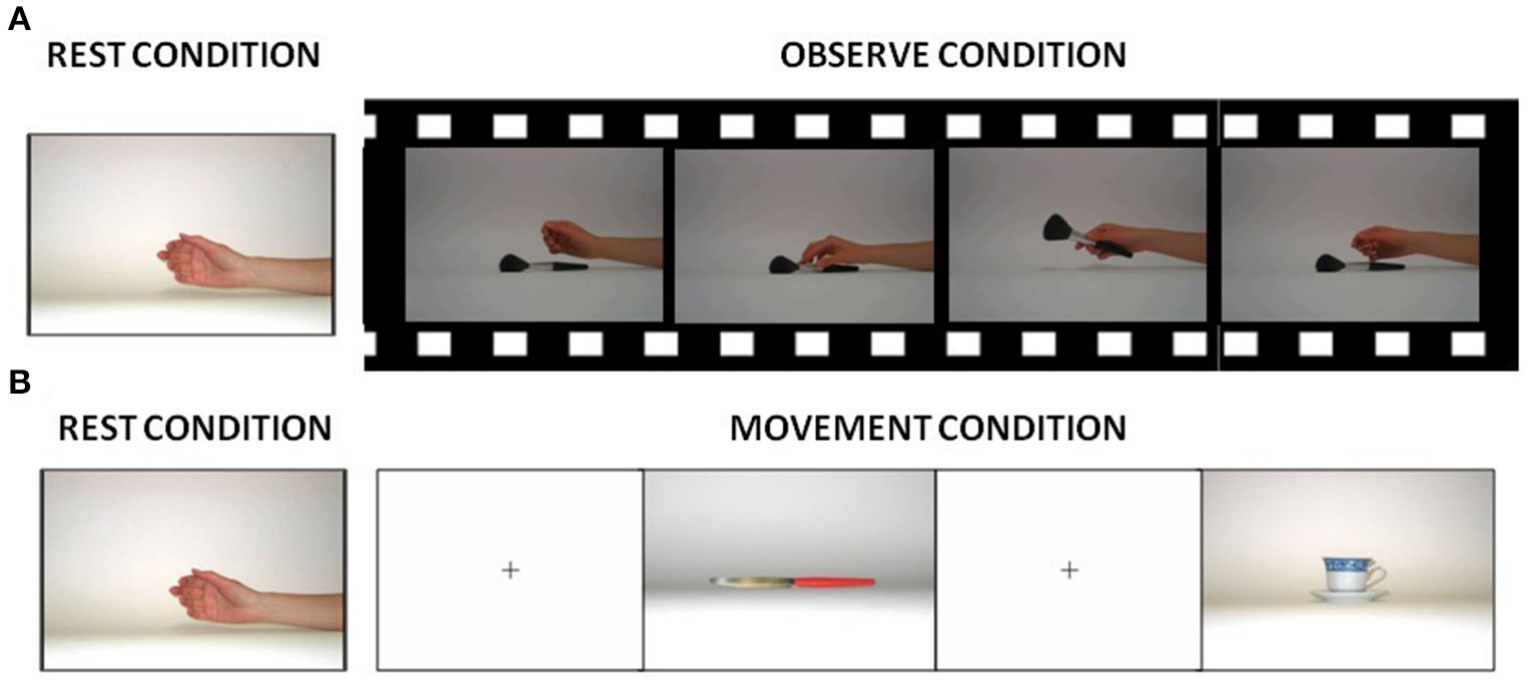
Visual stimuli presented to the subjects in each experimental condition. **(A)** Picture used during rest conditions and samples of frames from one of the videos presented to the subjects during the observation condition. **(B)** Pictures shown to the subjects during the rest and execution condition.

During the fMRI scanning, participants were asked to perform 2 block-designed runs (A-B structure): in one run, they were instructed to observe (observational run−s) and in the remaining run to execute hand grasping movements (motor run), according to a paradigm described in Cabinio et al. (2010). Briefly, in the observation run, all subjects viewed movies of hand grasping different objects (see Figure 1A). In the motor run, subjects observed visual stimuli consisting of objects oriented in order to be grasped with the right hand and they were asked to perform a grasping movement appropriate to the shape of the object (see Figure 1B; subjects were asked to perform movements as if they had the observed object actually in their hand, therefore the execution condition was a mime of the action). The motor act was performed continuously during the observation of the image of each object, with a frequency of about 1 Hz and a total number of 27 objects. Examples of objects were a cup, scissors, a screwdriver, and a torch. Figure 1 shows some visual stimuli presented to the subjects in each experimental condition. The decision to use different types of grasp (both observed and executed) was driven by the need to recruit the highest percentage of mirror neurons and thus increment the BOLD signal (Ehrsson et al., 2000; Iacoboni et al., 2005; Cabinio et al., 2010; Marino and Ricciardelli, 2017). Before the fMRI experiment, the participants were verbally instructed to execute the movements as if the object was close to their hand, and to carry out the task only with the hand and the wrist: repeating this action, until the appearance of the next picture.

Participants were trained outside the scanner for 15 min before data acquisition. During the training session, subjects were instructed to keep their gaze on the fixation point for the entire duration of the experiment, and to execute grasping movements about once every 2 s. If the training task (that was conducted with different parallel versions), was not properly executed by the subjects, they were not included in the study.

We used an MR-compatible visual system to present the stimuli (VisuaStim Digital system, Resonance Technology Inc.). The use of E-Prime software (E-Prime 2.0 Psychology Software tool, http://www.pstnet.com) ensured exact timing of prompts during MR acquisition. The performance was visually checked by the examiner during the execution of the task. The examiner maintained accuracy by reporting all the tasks correctly performed. The design was fully randomized (both blocks and runs). All the stimuli were projected onto a screen at the front side of the magnet bore and subjects viewed them through a mirror attached to the head coil.

#### MRI acquisition

Functional images were collected by a gradient echo-planar (EPI) T2^*^ sequence (TR = 3,000 ms; TE = 50 ms; flip angle = 90°; voxel size = 2,8125 × 2,8125 × 4 mm; matrix size = 448 × 448; number of slices = 38; thickness = 4 mm) using BOLD (blood oxygenation level dependent) contrast. Each fMRI session included two runs of 122 dynamics.

MRI data was acquired on a 1.5 Tesla Siemens Magnetom Avanto at Santa Maria Nascente Institute IRCCS, Don Carlo Gnocchi Foundation.

A conventional T2-weighted scan (TR = 2,920 ms; TE = 108 ms; voxel size = 0.75 × 0.75 × 5.2 mm; matrix size = 320 × 320; slice thickness = 4 mm; number of slices = 25) was performed in every subject in order to exclude brain abnormalities. A 3D T1-weighted FFE scan (TR = 1,900 ms; TE = 3.37 ms; voxel size = 1 × 1 × 1 mm; matrix size = 192 × 256; slice thickness = 1 mm; number of slices = 176) was acquired to be used as an anatomical reference for fMRI analysis and to compute hippocampal volumes on every subject.

#### Hippocampal segmentation

Bilateral hippocampi were segmented using a dedicated software (FSL-FIRST Patenaude et al., 2011). Brain tissue volume, normalized for subject head size, was estimated with SIENAX (Smith et al., 2002), part of FSL (Smith et al., 2004). An ANCOVA analysis was performed to compare hippocampal volumes between groups. The Scaling Factor, obtained using SIENAX, was included as a covariate in the analysis. Results were considered as statistically significant if surviving *p* < 0.05 with Bonferroni correction for multiple comparisons.

#### fMRI analysis

fMRI data was analyzed in agreement with the General Linear Model running on MATLAB 7.6 (MathWork, Natick, MA) and SPM12 (Wellcome Dept. Cogn. Neurol., London; http://www.fil.ion.ucl.ac.uk/spm). Images were first corrected for motion, then they were realigned and movement parameters were estimated. Anatomical and functional images were then spatially normalized to the MNI template using a 2 × 2 × 2 voxel size with a trilinear algorithm. The normalized functional images were spatially smoothed using a 8-mm full-width at half-maximum isotropic Gaussian kernel.

At the first-level, we modeled the expected hemodynamic response function of the software package with a block design. Six parameters related to head movement during scanning, were included as regressors of no interest. For each subject, we estimated two t-contrasts: observation of a hand grasping (O) and execution of grasping movements (M).

All contrasts defined in the First Level were included in the Second Level analysis of the three groups: NE, MCI, and AD.

In order to identify MN areas active during both movement, observation and execution, we performed on each group a conjunction analysis, between O and M contrasts. We exclude from the analysis, voxels not active in both contrasts at a certain threshold. To this aim, we masked the conjunction analysis with voxels active in both contrasts used to perform the conjunction at a threshold of *p* < 0.05, as done elsewhere (Cabinio et al., 2010; Cerri et al., 2015). We also included in the model, age, and MMSE value as nuisance covariates. With this analysis, we determined voxels active during both observation and execution of hand movements. The assumption is that neurons involved in the MN system are among those activated during both conditions. Thus, the conjunction analysis can be considered as a mask through which only those voxels that are significantly active in the two conditions are selected (O, M), even if they have different p values (provided that the p values are above the selected threshold). Moreover, one-way analysis of variance (ANOVA) was used to determine whether there were any significant differences between the three groups (NE, MCI, AD) in the single experimental conditions (O, M) including age and MMSE value as nuisance covariates in the GLM. F-Contrasts tested for the difference between the three groups and for mean activations in the entire sample. All these second-level results have been considered as statistically significant if surviving a threshold of *p* < 0.05 FWE-corrected with a k = 50 contiguous voxels.

### Neuropsychological assessment

The neuropsychological assessment was centered on functions considered to be linked to the MN system, such as language (particularly action naming; Kemmerer et al., 2012) and empathy; we also tested memory (as memory deficits characterize AD and aMCI) and attention/executive functions (abilities which are associated to frontal lobe functioning) (see Table 2 for test list). Subjects were all right-handed in according to the Edinburgh Handedness Inventory (Oldfield, 1971).

**Table 2:** List of Neuropsychological tests.

**Test**	**Acronym**	**Description**	**References**
Mini mental state examination	MMSE	The most popular instrument to screen dementia and quantify global cognitive level	Folstein et al., 1975; Measso et al., 1993
Free and cued selective reminding test	FCSRT	A test of verbal memory which controls for individual differences in attention and cognitive processing through a “controlled learning” study procedure in which the examinee searches for study items in response to a category cue. The category cues are then used to facilitate the recall of items not retrieved during a free recall test phase of the task. The FCSRT has been used to identify MCI, and distinguish AD from other types of dementia	Grober et al., 1987; Frasson et al., 2011
Immediate free recall	FCRST-IFR		
Immediate total recall	FCRST-ITR		
Delayed free recall	FCRST-DFR		
Delayed total recall	FCRST-DTR		
Cueing sensitivity index	FCRST-CSI		
Phonemic fluency	PF	Assessing the timed production of words after phonemic cues.	Novelli et al., 1986
Semantic fluency	SF	Assessing the timed production of words after semantic cues.	Novelli et al., 1986
Trail making test	TMT	Assessing cognitive abilities such as visual scanning and visual-motor tracking (Part A), executive function, visuo-conceptual function, visuo-motor tracking and sustained attention (Part B)	Giovagnoli et al., 1996
Part A	TMT-A		
Part B	TMT-B		
Repeatable battery for assessment of neuropsychological status naming test	RBANS-N	The naming test taken from the Repeatable Battery for Assessment of Neuropsychological Status, a brief battery which measures immediate and delayed memory, attention, language, and visuospatial skills	Randolph et al., 1998; Ponteri et al., 2007
Revised reading the mind in the eyes	RRME	Testing the ability of inferring others' mental states. Performance on this test correlates with an empathic personality	Baron-Cohen et al., 2001; Vellante et al., 2013
Action naming	AN	An *ad-hoc* task to test of action naming, a function considered to be linked to MN	Spada, 2012

## Results

### Hippocampal volume measurements

The ANCOVA analysis showed statistically significant differences in bilateral hippocampal volume between the NE and the MCI groups, as well as between the Control and the AD groups. No volumetric differences were observed between the MCI and the AD groups. Differences were found in both Left and Right Hippocampi (see Table 3).

**Table 3:** Hippocampal (HP) volumetric comparison between the three groups.

	**2**	**3**	**1**		
**HP Volume**	**NE**	**MCI**	**AD [MM**+**SdE]**		***Post-hoc*** **(Bonferroni)**
Left HP [MM, SdE]	3521.6542 [123,1601]	2872.3732 [126,4296]	2791.97 [125.35]	^*^p < 001	1 vs. 2, 2 vs. 3
Right HP [MM, SdE]	3718.7444 [111,7622]	3086.8005 [108,872]	2763.1426 [110,804]	^*^p < 001	1 vs. 2, 2 vs. 3

*MM, marginal mean; SdE, standard Error; NE, Normal Elderly people; MCI, Mild Cognitive Impairment; AD, Alzheimer Disease*.

### fMRI conjunction analysis

**Figure 2:**
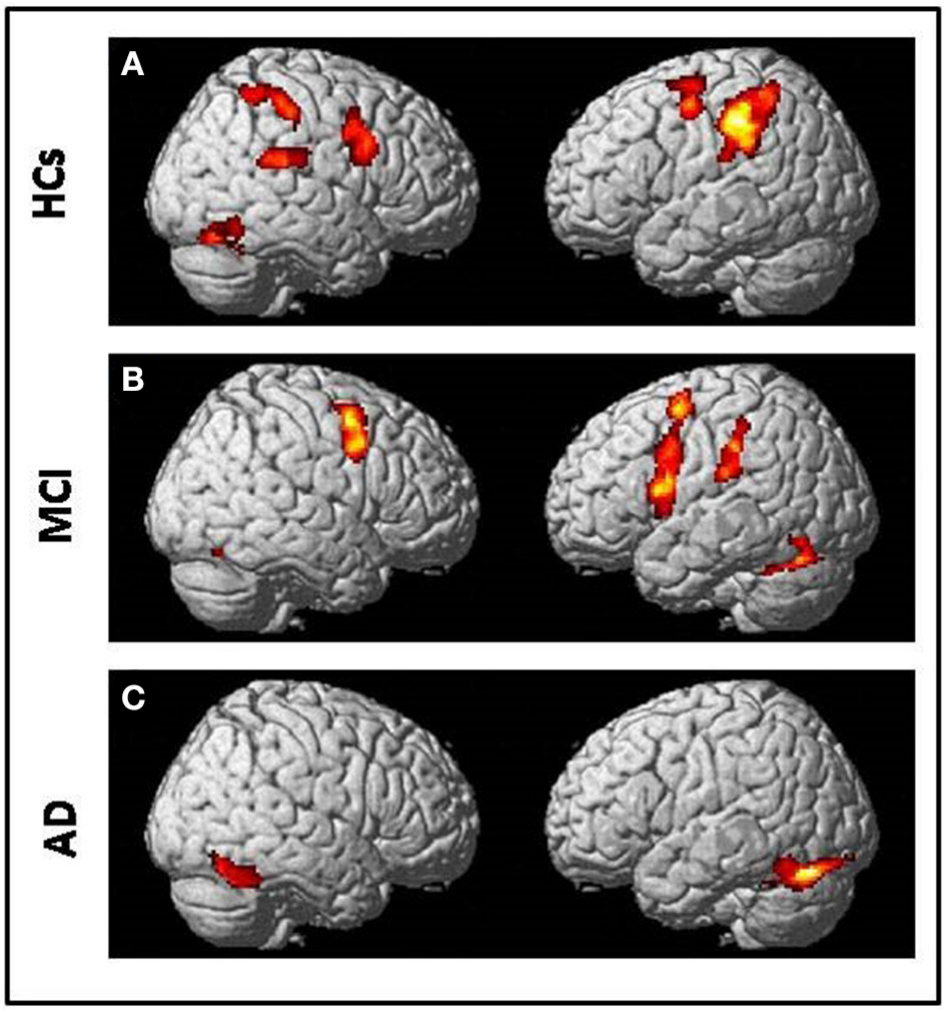
Conjunction analysis (comparing fMRI activation during observation of transitive hand grasping and execution of grasping movements) results respectively in **(A)** Normal elderly (HCs) group; **(B)** Mild Cognitive Impairment (MCI) group and **(C)** Alzheimer's Disease (AD) group [t contrast 3.41; k = 50; pcluster(FWE-corr) < 0.05 only].

All subjects correctly performed the fMRI runs. Given the premise that regions included in the MN network must be activated both in movement execution and in movement observation, to identify cortical areas involved in this network we performed conjunction analysis between activation of the two conditions M and O. This analysis was carried out separately for the three groups of NE, MCI, and AD subjects. All regions of significantly increased activation (pFWE < 0.05 at cluster level with K = 50 contiguous voxels) are summarized in Table 4 and illustrated in Figure 2, each group separately.

**Table 4:** Conjunction analysis results respectively, in NE, MCI, and AD.

**Cluster**	**Cluster level**		**Z_score**	**Coordinates**			**Brain Region [BA]**
**[k]**	**p FWE-corr**	**p uncorr**		**x,y,z [mm]**			
**[A] NE**							
1,719	0.000	0.000	5.48	−42	−36	42	L Inferior Parietal Lobule [40]
			5.14	−36	−38	48	
			4.75	−50	−30	42	
591	0.002	0.000	4.79	40	4	28	R Precentral Gy [9 / 6]
			4.37	46	12	30	
			4.11	46	4	40	
383	0.013	0.002	4.73	50	−34	54	L Inferior Parietal Lobule [40]
			4.14	58	−28	48	
			3.81	40	−46	58	
361	0.017	0.002	4.54	−42	−6	50	L Precentral Gy [6]
			3.75	−32	−6	64	
			3.60	−32	2	62	
292	0.038	0.005	4.14	62	−40	22	R Inferior Parietal Lobule [40]
			4.06	68	−24	22	
			3.57	60	−32	20	
351	0.019	0.003	4.05	32	−54	−22	R Fusiform Gy
			4.04	60	−60	−14	
			4.03	38	−72	−20	
**[B] MCI**							
506	0.004	0.001	5.49	38	2	58	R Precentral Gy [6 / 9]
			5.08	46	4	36	
			4.06	44	2	46	
1060	0.000	0.000	5.03	−36	0	58	L Precentral Gy [6]
			4.47	−50	4	14	L Inferior Frontal Gy [44]
			4.41	−36	6	20	
271	0.047	0.007	4.50	30	−60	−18	R Cerebellum / Fusiform Gy
			4.19	28	−44	−24	
326	0.025	0.004	4.04	−52	−66	−14	L Middle Occipital Gy / Fusiform Gy
			4.02	−42	−70	−22	L Cerebellum
			3.99	−34	−52	−26	
332	0.023	0.003	4.00	−62	−28	26	L Inferior Parietal Lobule [40]
			3.78	−56	−24	22	
			3.53	−52	−34	48	
**[C] AD**							
831	0.000	0.000	5.16	36	−62	−18	R Fusiform Gy [37]
			4.45	44	−46	−20	
			4.34	44	−54	−24	
1222	0.000	0.000	4.91	−40	−72	−22	L Cerebellum
			4.75	−34	−48	−24	
			4.58	−30	−72	−20	L Fusiform Gy [37]

*R, right; L, left; BA, Brodmann area; Gy, Gyrus; NE, Normal Elderly people; MCI, Mild Cognitive Impairment; AD, Alzheimer Disease. Coordinates are expressed in MNI space; statistical values refer to the conjunction analysis in [A] NE, [B] MCI, [C] AD [t contrast 3.41; k = 50; pcluster (FWE-corr) < 0.05]*.

In NE subjects the conjunction analysis showed a statistical significant [t contrast 3.41; k = 50; pcluster (FWE-corr) < 0.05] activation of the bilateral fronto-parietal network (left>righ side) formed by the frontal cortex (Precentral Gyrus, PrCG, BA6) and the parietal cortex (Inferior Parietal Lobule, IPL, BA40) along with activation of the right STG (BA22/42) and the right Fusiform Gyrus (FuG).

In MCI subjects the same conjunction analysis [t contrast 3.41; k = 50; pcluster(FWE-corr) < 0.05] revealed the activation of the frontal-parietal network (right < left side) including also the left Inferior Frontal Gyrus (IFG, BA44), but activation of BA40 was not as strong as in NE participants. A bilateral cerebellar activation was also detected (See Table 4).

Finally, in AD patients the conjunction analysis [t contrast 3.41; k = 50; pcluster (FWE-corr) < 0.05] showed a statistical significance recruitment in the bilateral FuG and in the left cerebellum. It found no activations in the fronto-parietal network.

**Figure 3:**
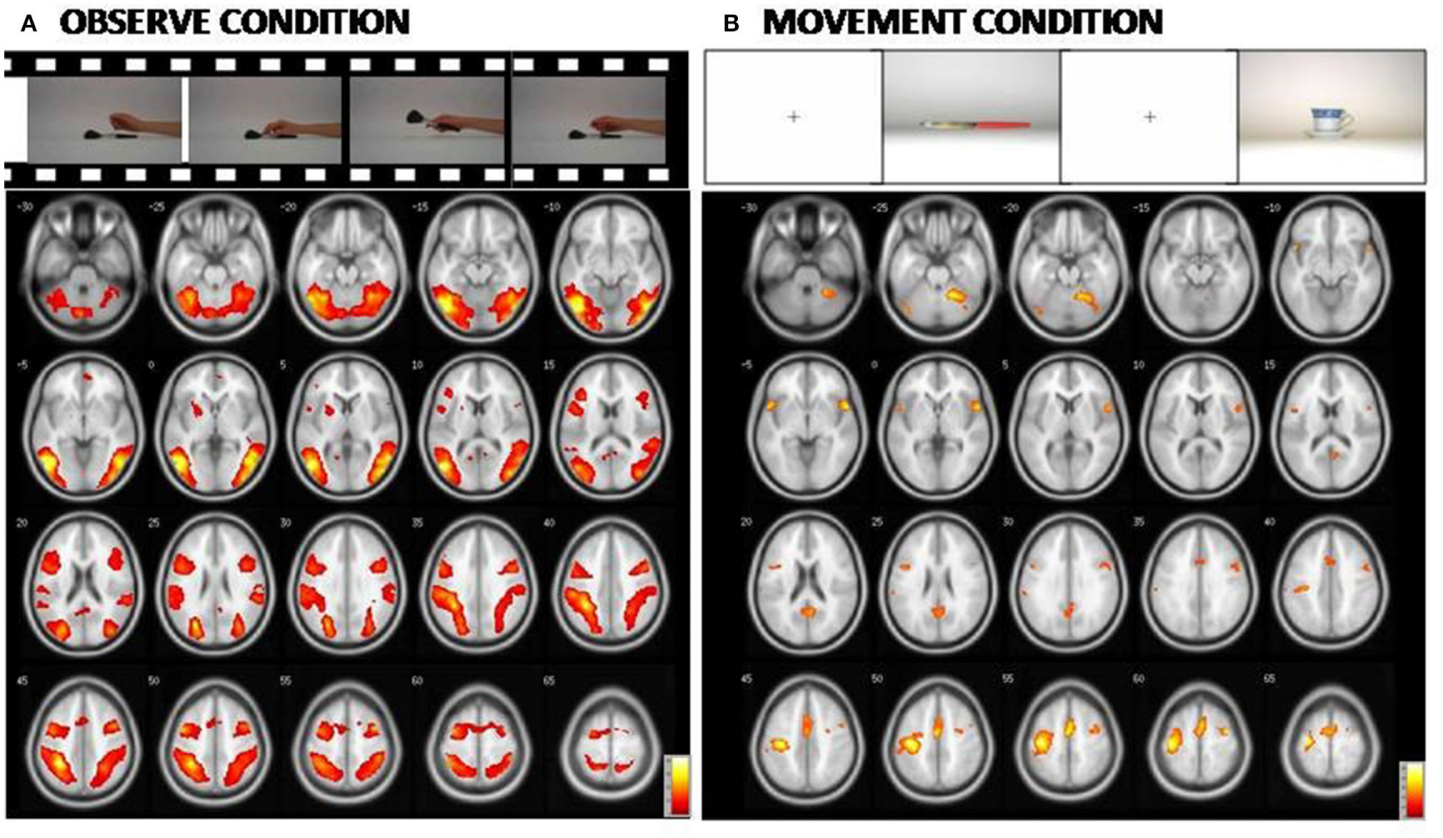
At the **(A)**: Representation of the cerebral areas involved in the Observed condition vs. the Rest condition in the three groups (NE, MCI, and AD). At the **(B)**: Representation of the cerebral areas activated in the Movement condition vs. the Rest condition in the three groups (NE, MCI, and AD). In the Observed condition, visual areas are also highly activated. [Main effect of fMRI task: **(A)** Observed condition (F contrast 14.74; k = 50; p(FWE-corr) < 0.05); **(B)** Movement condition (F contrast 13.48; k = 50;p(FWE-corr) < 0.05)].

An additional analysis to investigate potential differences in brain activation between the three groups for the M or O condition was carried out separately, and did not reveal any significant group differences. The Figure 3 and Table 5 report the statistical results of the main effect in the O condition [ANOVA, F contrast 14.74; K = 50; p(FWE-corr) < 0.05] and in the M condition [ANOVA, F contrast 13.48; k = 50; p(FWE-corr) < .05].

**Table 5:** Main effect of fMRI task in the Observe run and Movement run.

**Cluster**	**Z_score**	**Coordinates**			**Brain Region [BA]**
**[k]**		**x,y,z [mm]**			
**OBSERVE vs. REST**					
7,451	7.68	−42	−70	−12	L MTG / MOG
	7.58	−34	−44	46	L IPL [40]
	7.49	−46	−72	10	
3,617	7.34	50	−68	−8	R MTG / MOG
	6.97	44	−64	2	
	6.96	58	−60	6	
549	6.83	38	2	58	R PrCG [9 / 6]
	6.13	40	6	48	
	5.86	46	4	40	
935	6.79	−44	0	50	L PrCG [9 / 6]
	6.33	−34	−2	60	
	5.80	−48	4	34	
736	6.20	44	−38	58	R IPL [40]
	5.96	28	−54	44	
	5.42	38	−50	56	
369	6.11	−4	−78	−34	L cbl
	5.61	8	−80	−36	R cbl
	5.43	−12	−76	−46	
**MOVEMENT vs. REST**					
1670	7.11	−40	−20	54	L PrCG / PoCG [6 / 4]
	6.69	−32	−18	66	
	5.29	−56	−30	50	
350	6.64	54	14	−8	R IFG
600	6.52	18	−52	−22	R cbl
	5.24	38	−72	−22	
151	6.36	−50	16	−8	L IFG
1,081	6.33	−2	−6	52	L SMA [6]
	6.12	2	6	58	
	5.75	−4	−6	66	
209	6.10	36	−6	60	R MFG [6]
	5.51	24	−2	64	
	5.03	28	−4	48	
65	5.86	−42	−72	−22	L cbl
396	5.66	4	−58	20	R PC [23]
	4.70	2	−44	30	
207	5.33	48	4	46	R PrCG [9 / 6]
	5.25	54	4	38	
	5.06	46	8	30	
52	5.28	−64	−26	24	L IPL [40]
	4.85	−62	−26	36	

*R, right; L, left; BA, Brodmann area; MFG, Middle Frontal Gyrus; IFG, Inferior Frontal Gyrus; PrCG, Precentral Gyrus; PoCG, Post Central Gyrus; SMA, Supplemental Motor Area; PC, Posterior Cingulate; IPL, Inferior Parietal Lobule; MTG, Middle Temporal Gyrus; MOG, Middle Occipital Gyrus; cbl, cerebellum; NE, Normal Elderly people; MCI, Mild Cognitive Impairment; AD, Alzheimer Disease*.

*Coordinates are expressed in MNI space; statistical values refer to the main effect of Observe condition [ANOVA, F contrast 14.74; k = 50] and Movement condition [ANOVA, F contrast 13.48; k = 50], at statistical threshold voxel-wise pFWE-corrected < 0.05*.

### Neuropsychological tests

The results of AD patients were worse than NE subjects in all neuropsychological tests. These tests comprised tests of action naming and empathy, functions attributed to MN network. The only exception was the object naming test (probably due to a ceiling effect). aMCI subjects were significantly different from NE participants only in the episodic memory test, the FCRST, and in semantic fluency (see Table 6 for results).

**Table 6:** Neuropsychological tests—Group comparison (ANOVA).

	**NE**	**MCI**	**AD**	***P*§**	***Post-hoc* (Bonferroni)**	NE vs. MCI^*^	NE vs. AD^*^	MCI vs. AD^*^
#	16	16	16					
MMSE	28.8 ± 0.9	27.0 ± 2.0	22.4 ± 3.5	<0.001	NE > MCI > AD	−**1.161 (**−**2.22:**−**0.101)**	−**2.505 (**−**3.813:**−**1.196)**	−**1.614 (**−**2.742:**−**0.485)**
FCSRT—IFR	29.0 ± 3.5	19.2 ± 6.0	10.3 ± 7.3	<0.001	NE > MCI > AD	−**1.195 (**−**3.195:**−**0.796)**	−**3.267 (**−**4.764:**−**1.77)**	−**1.332 (**−**2.415:**−**0.249)**
FCSRT—ITR	36.0 ± 0.0	34.9 ± 2.0	27.2 ± 7.5	<0.001	NE > AD, MCI > AD	*−0.778 (−1.794:0.239)*	−**1.659 (**−**2.796:**−**0.523)**	−**1.403 (**−**2.497:**−**0.309)**
FCSRT—DFR	9.8 ± 1.7	5.9 ± 3.3	2.9 ± 3.2	<0.001	NE > MCI > AD	−**1.486 (**−**2.593:**−**0.379)**	−**2.693 (**−**4.046:**−**1.34)**	*−0.923 (−1.954:0.108)*
FCSRT—DTR	11.9 ± 0.5	10.7 ± 2.4	7.3 ± 3.6	<0.001	NE > AD, MCI > AD	*−0.692 (−1.701:0.317)*	−**1.79 (**−**2.95:**−**0.63)**	−**1.111 (**−**2.164:**−**0.058)**
FCSRT—CSI	1.0 ± 0.0	0.94 ± 0.09	0.68 ± 0.25	<0.001	NE > AD, MCI > AD	*−0.943 (−1.976:0.09)*	−**1.81 (**−**2.974:**−**0.647)**	−**1.384 (**−**2.475:**−**0.293)**
PF	37.9 ± 9.6	31.8 ± 9.6	20.4 ± 7.0	<0.001	NE > AD, MCI > AD	*−0.635 (−1.64:0.369)*	−**2.131 (**−**3.358:**−**0.904)**	−**1.405 (**−**2.499:**−**0.31)**
SF	39.6 ± 10.4	31.7 ± 9.2	17.6 ± 5.7	<0.001	NE > MCI > AD	*−0.805 (−1.823:0.214)*	−**2.623 (**−**3.96:**−**1.287)**	−**1.842 (**−**3.012:**−**0.673)**
TMT-A	45.2 ± 19.3	53.0 ± 14.6	88.4 ± 43.0	<0.001	NE < AD, MCI < AD	*0.456 (−0.537:1.448)*	**1.296 (0.218:2.374)**	**1.102 (0.051:2.154)**
TMT-B	95.6 ± 48.3	175.9 ± 106.6	391.1 ± 148.1	<0.001	NE < AD, MCI < AD	*0.97 (−0.066:2.006)*	**2.683 (1.332: 4.033)**	**1.668 (0.53:2.806)**
RBANS-N	10.0 ± 0.0	9.7 ± 0.9	8.8 ± 1.8	0.019	–	*−0.471 (−1.465:0.522)*	*−0.943 (−1.976:0.09)*	*−0.632 (−1.637:0.372)*
RRME	21.3 ± 4.0	18.4 ± 4.9	12.2 ± 4.7	<0.001	NE > AD, MCI > AD	*−0.648 (−1.654:0.357)*	−**2.085 (**−**3.303:**−**0.868)**	−**1.291 (**−**2.369:**−**0.214)**
AN	35.3 ± 3.6	31.6 ± 4.7	22.8 ± 6.4	<0.001	NE > AD, MCI > AD	*−0.884 (−1.911:0.143)*	−**2.407 (**−**3.694:**−**1.12)**	−**1.567 (**−**2.688:**−**0.447)**

§ *ANOVA (non- parametric tests -Kruskal—Wallis—give the same results)*.

* *In **bold**, are shown statistically significant pairwise comparisons. In italics, are shown effect size (Cohen's d – 95% CI): < 0.2: no effect; 0.2 to 0.5: small effect; 0.5 to 0.8: intermediate effect; 0.8 and higher: strong effect*.

*NE, Normal Elderly people; MCI, Mild Cognitive Impairment; AD, Alzheimer Disease. For test acronyms, see Table 2*.

## Discussion

We investigated the integrity of MN areas through fMRI in three groups: NE subjects, people with aMCI and AD patients. We detected differences among the three groups, suggesting a progressive weakening of the MN network with respect to neurodegenerative process. These differences are discussed in the following sections.

### MN network alteration

In the NE group, the conjunction analysis (fMRI activation recorded during observation of hand grasping compared to overt execution of grasping movements) showed the activation of a bilateral, though strongly left-lateralized, fronto-parietal network (PrCG, BA6; IPL, BA40). The fronto-parietal network activated in our study is considered part of the human MN network (Rizzolatti and Craighero, 2004), given the established homology between the BA6 (human) and the ventral premotor cortex (monkey), the BA40 (human), and PF-PFG (monkey) parietal cortex (Cook et al., 2014). We also found activation of the STG (BA22/42) considered an area which is strongly associated with activation of MN in humans (Aziz-Zadeh et al., 2006).

fMRI and neurophysiological investigations aimed to study the MN network in human were mostly performed on young or adult subjects. We found that the MN network is largely preserved in aging. Our results are in accordance with those of Léonard and Tremblay (2007), who analyzed corticomotor facilitation associated with observation, imagery and imitation of hand actions in younger and older adults by monitoring changes of motor evoked potentials elicited in hand muscles by TMS. They found that corticomotor facilitation in association with covert action execution was largely preserved with aging, although with a loss in selectivity for activated muscles. Unlike the original study of Cabinio et al. (2010), in this study (where we exactly replicated their procedure), we noted a loss of selectivity, as the activation of mirror areas was more bi-lateralized than in the younger subjects. This could be explained by a specific mode of brain activation in aging. In fact, fMRI recording in younger, middle-aged and older participants performing the same unilateral hand movements (Fang et al., 2005) showed stronger premotor/motor cortex activity in the contralateral hemisphere in the “older” group when compared to the younger and middle-aged groups. Therefore, together with other authors, we concluded that the older brain requires larger areas to achieve the same task (Fang et al., 2005; Chow et al., 2017), a hypothesis that could also explain the results obtained during a task inducing a motor resonance mechanism (Rizzolatti, 2005). However, in order to exclude any bias, due to differences in the scanners, it would be worthwhile recruiting a young subject pool to definitively confirm the data.

Concerning the MCI subjects, we found a different pattern at the conjunction analysis in comparison with the NE subjects. The MCI and the NE groups showed similar activation of frontal and parietal areas. However, in the MCI group the left IPL (parietal BA40) was activated at a lesser extent than in the NE group and neither the right IPL nor the STG (BA22/42) were activated. Moreover, only the MCI participants exhibited activation of the left frontal area IFG-BA44. Therefore, even if the MN network is preserved in MCI, it is mainly recorded toward frontal areas. This suggests a greater resilience to the aging process of the MN areas located in the anterior part of the brain vs. posterior areas, and maybe consecutively the capacity of these areas to take on a part of the “work” done by the posterior MN regions. Interestingly, we did not observe any activation of the fronto-parietal network in AD participants. Altogether, our results suggest that the MN network is progressively affected in neurodegenerative cognitive decline following a posterior-anterior gradient. The hypothesis of a posterior-anterior decay in Alzheimer-type degeneration agrees with recent data obtained in MCI using EEG and MRI recording (Moretti, 2016). In this investigation, alpha3/alpha2 frequency power ratio, considered as a predictor of conversion into AD (Moretti et al., 2011) as well as cortical thickness at MRI were computed. Three MCI groups were obtained considering increasing tertile values of alpha3/alpha2 ratio. From this morphological MRI and EEG parameter data the author inferred that MN are impaired in prodromal AD. Conversely, we directly measured the activation of human MN with a specific fMRI task, thus giving a functional support to the hypothesis for the MCI and the AD group.

### Motor cognition and neurodegenerative process

While unexpected, the present results raised several questions. How to explain the cerebellar activation recorded in AD patients and to a lesser extent in MCI subjects? Based on models incorporating the MN system in social learning, the STG-IPL-IFG circuit would create the motor representation available for imitation, starting with the visual input (Iacoboni et al., 1999; Oh et al., 2012). During the imitation phase, the overall processes are equivalent to those of the observation phase, the only difference is that, in parallel with the IFG to the IPL pathway, neural drive is sent to the musculoskeletal system through M1 to perform the action (Oh et al., 2012; Bassolino et al., 2013). It has been suggested that the cerebellum provides the prediction error for the IFG and the IPL to adjust internal models (Miall, 2003; Oh et al., 2012). In this context, the AD patient activation of cerebellar neurons involved in the MN network would correspond to an attempt to compensate for damage of prefrontal and parietal areas. This mechanism would already be present in aMCI. The activation of the cerebellum as a substitute of cerebral cortex alteration has also been suggested to enhance motor control and motor learning in functional recovery from a stroke following mirror therapy (Arya et al., 2017). In conclusion, cerebellar activation in AD might simply reflect the response of this neural structure to visual inputs during guided limb movements (Liu et al., 2003).

### Clinical implications and limitations

The alteration of the MN motor circuitry in AD patients raises an interesting question related to the link between action and cognition and supports an interesting direction of research. Precisely, it allows the interpretation of AD—as other neurodegenerative diseases (Bak, 2013; Eisen et al., 2014) in the light of the action-perception coupling hypothesis. In contrast to traditional views (considering motor mechanisms as a slave system of cognition), it is recurrently postulated that a part of cognition results from the coupling between action and perception representations, and corresponds to implicit action simulation (or “motor resonance”) instead of explicit recall of abstract symbols (Jeannerod, 1994, 2001, 2006; Jeannerod et al., 1995; Boulenger et al., 2008; Pulvermüller and Fadiga, 2010). Neuropsychological tests exploring cognitive functions that considered to be tightly linked to the MN system (language and empathy) were normal in aMCI subjects. The preservation and maybe hyperactivation of the anterior MN network presently seen in aMCI, might supplement the initial decay of the posterior part of the neural circuit, thus preserving cognitive performance. The only “language” test impaired in aMCI was semantic fluency, but this impairment could be due to semantic memory damage, rather, than true language impairment. As an alternative explanation, the preservation/hyperactivation of the anterior MN network could not be sufficient to support performance in this kind of task because it is highly dependent on parietal areas (Seghier, 2013). In contrast, in the AD group the MN network appears to be deficient as task performance in both language and empathy tests deteriorates.

The present study is limited by several factors: the number of subjects tested and the spatial and temporal resolution of our measurements (see Oosterhof et al., 2011, 2013; Möhring et al., 2014; Thibault and Raz, 2016); the fact that in the execution condition the hand movements were only visually controlled by the examiner. In particular, our results should now be replicated on the basis of data collected with different methods dedicated to record MN activity, as motor evoked potentials induced by TMS (Gangitano et al., 2004; Fadiga et al., 2005), or the reduction of magnitude of the mu rhythm at EEG (Fox et al., 2016 for a review). Furthermore, we have studied task-evoked neural activity during action observation and execution. Schilbach et al. (2016), in a recent study, have demonstrated differential patterns of dysconnectivity in MN and mentalizing networks in schizophrenia. Further research, therefore, needs to investigate functional organization of self-related brain networks during a “resting state.” This clinically available measure of functional connectivity could provide further knowledge of alterations of neurofunctional systems in AD.

The results of our study have possible implications in rehabilitation. Recently, the use of action observation as a supplementary therapeutic tool for patients who have had strokes, in order to stimulate brain plasticity and obtain positive functional results, has been reported (see Carvalho et al., 2013 for a review on this topic). This has been extended to motor deficits of children with Cerebral Palsy (Sgandurra et al., 2011; Buccino et al., 2012; Bassolino et al., 2015) and patients with Parkinson's disease (Buccino et al., 2011). The concept of motor cognition, if verified, would provide a clinical support to cognitive stimulation based on the motor resonance mechanism. However, the efficiency of such training is only conceivable if the MN system remains partly achievable. So far, the only published research of this kind of cognitive training via the stimulation of the MN in people with AD, showed improvements in attention and facial recognition (Eggermont et al., 2009) whereas another research showed negative results (Caffarra, 2016, unpublished, oral communication at XI Sindem National Congress, Italy). However, this last study did not test MN system integrity, that is supposed to be stimulated in the training program. As our data showed a malfunction in the MN network in the case of AD, our study explains why that intervention obtained negative results. A rehabilitative intervention based on the MN system would be better implemented at the MCI phase.

## Author contributions

EF contributed to the conception and design of the research along with the interpretation of data, wrote the main draft, and oversaw the revision of the work; FB contributed to the conception and design of the work, performed the analysis and interpretation of neuroimaging data and helped in drafting the work; SP analyzed neuropsychological data and helped in drafting the work; AD acquired most neuropsychological data and contributed to their analysis; IC contributed to the acquisition of data and in drafting the work; SD contributed to the acquisition of neuroimaging data and to the analysis of neuroimaging and neuropsychological data; GB contributed to the acquisition of data for the work and in drafting the work; TP contributed to the conception and design of the work, interpretation of data and in writing the paper. All the Authors approved the final version of the work to be published and agreed to be accountable for all aspects of the work.

### Conflict of interest statement

The authors declare that the research was conducted in the absence of any commercial or financial relationships that could be construed as a potential conflict of interest.
